# Time-Resolved Structural Measurement of Thermal Resistance across a Buried Semiconductor Heterostructure Interface

**DOI:** 10.3390/ma16237450

**Published:** 2023-11-30

**Authors:** Joohyun Lee, Wonhyuk Jo, Ji-Hwan Kwon, Bruce Griffin, Byeong-Gwan Cho, Eric C. Landahl, Sooheyong Lee

**Affiliations:** 1 Korea Research Institute of Standards and Science, Daejeon 34113, Republic of Korea; joohyun.lee@kriss.re.kr (J.L.); kwonjh@kriss.re.kr (J.-H.K.); newbgcho@gmail.com (B.-G.C.); 2 European X-ray Free-Electron Laser GmbH, 22869 Schenefeld, Germany; wonhyuk.jo@xfel.eu; 3 Department of Nano Convergence Measurement, Korea University of Science and Technology, Daejeon 305-340, Republic of Korea; 4 Department of Physics and Astrophysics, DePaul University, Chicago, IL 60614, USA; ffbjgriffin@yahoo.com (B.G.); elandahl@depaul.edu (E.C.L.)

**Keywords:** time-resolved X-ray diffraction, thermal transport, semiconductor, interface, nanoscale, heterostructure

## Abstract

The precise control and understanding of heat flow in heterostructures is pivotal for advancements in thermoelectric energy conversion, thermal barrier coatings, and efficient heat management in electronic and optoelectronic devices. In this study, we employ high-angular-resolution time-resolved X-ray diffraction to structurally measure thermal resistance in a laser-excited AlGaAs/GaAs semiconductor heterostructure. Our methodology offers femtometer-scale spatial sensitivity and nanosecond time resolution, enabling us to directly observe heat transport across a buried interface. We corroborate established Thermal Boundary Resistance (TBR) values for AlGaAs/GaAs heterostructures and demonstrate that TBR arises from material property discrepancies on either side of a nearly flawless atomic interface. This work not only sheds light on the fundamental mechanisms governing heat flow across buried interfaces but also presents a robust experimental framework that can be extended to other heterostructure systems, paving the way for optimized thermal management in next-generation devices.

## 1. Introduction

The manipulation of microscopic heat flow in heterostructures has garnered significant scientific attention given its vast potential in diverse applications ranging from thermoelectric energy conversion [1,2,3,4] to high-efficiency thermal barrier coatings [5,6] and precise heat dissipation in electronic [7] and photovoltaic devices [8]. As technological advancements propel the miniaturization of high-power-density devices, the imperative for efficient thermal load management becomes increasingly evident. Such management is not only crucial for the longevity and reliability of these devices but also for their optimal performance [9].

The burgeoning demand for intricate device designs has underscored the need for non-invasive methodologies capable of probing heat transport at buried interfaces. Traditionally, Fourier’s law of heat diffusion has served as the cornerstone for understanding thermal transport phenomena in bulk materials [10,11,12]. This classical model posits a continuous thermal gradient within the local equilibrium. However, recent studies have cast doubts on its universal applicability, especially under certain conditions. Notably, when the characteristic dimensions of devices approach the mean free path (MFP) of dominant heat carriers, such as electrons and phonons, or when there is a significant increase in heterostructure interfaces, deviations from Fourier’s law become apparent [12,13,14,15]. Such deviations have been corroborated by a plethora of experimental and theoretical investigations, which have reported significant disparities in thermal resistance values across various nanostructures [16], superlattices [17,18], nanowires [19], and porous films [20]. These findings have ignited discussions on the role of ballistic long-wavelength phonons in heat transport. In many of these scenarios, even the seemingly simple task of measuring the temperature on either side of an interface without perturbing the system’s temperature becomes a formidable challenge [21,22,23]. Consequently, the role of phonons in non-steady-state heat transport and their relationship with interfacial thermal resistance remains an enigma. A significant impediment to a deeper understanding is the current limitation in measuring structural changes at the requisite lengths and timescales. These scales are essential for tracking heat propagation across buried interfaces, which can span from tens of nanometers to several micrometers [24,25,26,27].

In recent years, the femtosecond laser has emerged as an invaluable instrument for generating transient heat sources, enabling researchers to delve into the thermal properties of materials in non-steady states [28,29,30]. The time-domain thermoreflectance (TDTR) method, in particular, has gained widespread acceptance for its ability to measure the thermal resistance of constituents and interfaces in heterostructures [31,32,33,34]. This technique hinges on observing the surface reflectivity changes of a sample post-illumination by femtosecond laser pulses. The ensuing time-dependent reflectance is subsequently juxtaposed with the two-step thermal relaxation model, which predicates a linear correlation between reflectivity and temperature [35,36]. Another noteworthy method is Raman thermometry, which has demonstrated its efficacy in gauging temperature fluctuations in atomistically thin materials [37,38,39]. However, a significant limitation of these optical techniques is their confinement to surface dynamics, restricted by the penetration depths of light. This constraint often results in a dearth of insights into structural alterations in buried interfaces or the bulk of materials. Moreover, the all-optical responses can sometimes produce amalgamated contributions from neighboring layers in a multi-layered specimen, further complicating the analysis [40]. Electrothermal methods, exemplified by the 3ω technique, grapple with analogous challenges when probing thermal transport in buried interfaces. The absence of depth-specific sensitivity in these methods obscures the individual contributions from the bulk material and interface, rendering it arduous to distinguish their distinct thermal attributes [41,42,43].

In contrast, time-resolved X-ray diffraction (TRXD) is a powerful technique that provides insights into the lattice dynamics within individual layers of heterostructures and materials beneath opaque capping layers [44,45,46,47]. The depth of hard X-rays into solids, combined with material-specific Bragg diffraction conditions, makes TRXD a valuable tool. One of the key strengths of TRXD lies in its sensitivity to depth-dependent strain in nearly perfect crystals. This sensitivity is manifested through changes in the shape of diffraction peaks, which are influenced by dynamical diffraction effects. These effects, resulting from the interaction of X-rays with the periodic potential of the crystal, provide a detailed view of strain distributions and lattice distortions, enabling precise measurements of structural dynamics at various depths within the crystal.

Recent advancements in synchrotron sources have enabled high-angular-resolution TRXD measurements, allowing researchers to study various transient phenomena such as impulsively driven collective lattice displacements [48,49,50,51], ballistic phonon heat propagation [48], and the generation of anisotropic strains [52]. Determining X-ray rocking curve shapes is essential, especially when considering their narrow widths. A temperature increase in bulk semiconductors can cause shifts in these curves, corresponding to strains on the order of $10^{-6}$ or smaller [53].

In this study, we utilized high-angular-resolution TRXD [53] to measure the structural thermal resistance in a laser-excited AlGaAs/GaAs semiconductor heterostructure. Our approach involved measuring heat-induced changes in diffraction patterns from the film, substrate, and their interface. During the X-ray measurements, we observed the effects of impulsively driven strains, evident as distortions in the X-ray interference fringe patterns between the primary diffraction curves of the film and substrate [46]. This effect persisted for up to 60 ns after the initial laser excitation. As the experiment progressed, we observed lattice relaxation due to heat transfer from the film to the substrate layer. The GaAs substrate experienced a gradual increase in temperature, with a maximum rise of about 5 K. We conducted a time-dependent strain analysis to determine the thermal resistance governing heat transport. This analysis helped ascertain the heat flux across the material interface and the temperature difference between the layers over time. The observed relationship between the direct measurements of heat flux and temperature gradient across the interface yields the effective thermal resistance across the heterostructure. The difference between our experimental results and the bulk reference values can be attributed to the thermal boundary resistance at the epitaxial interface, aligning with previous research [54,55]. Our findings support the applicability of Fourier’s Law in describing heat transfer from thin films to bulk substrates, especially when thermal transport is primarily diffusive [9].

## 2. Materials and Methods

**Figure 1:**
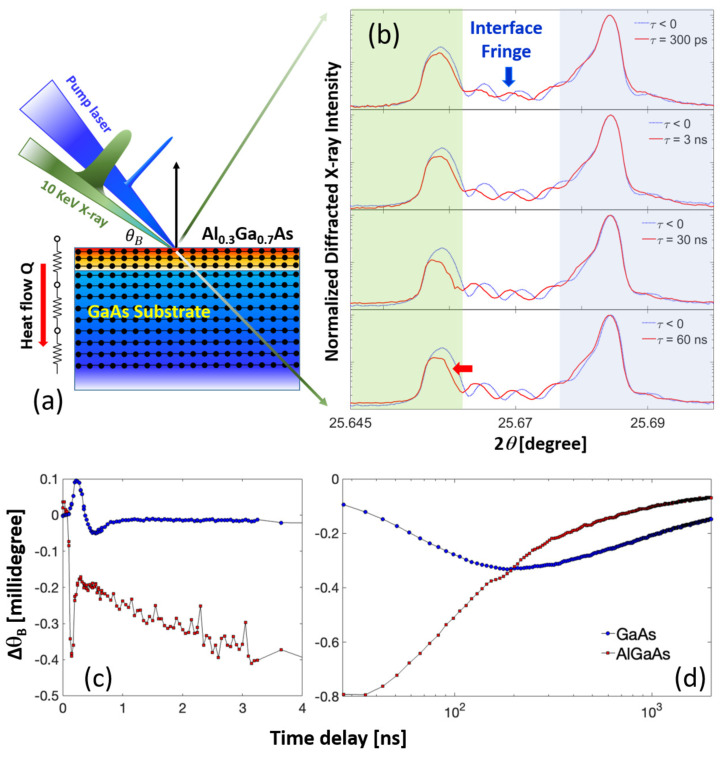
(**a**) Schematic of the TRXD experiment of a single heterostructure sample consisting of a 500 nm (0 0 1) Al${}_{0.3}$Ga${}_{0.7}$As epitaxial film grown on (0 0 1) GaAs substrate. The two (0 0 4) Bragg peaks are clearly resolved. (**b**) Time- and angle-resolved intensity profiles of the Bragg diffraction peaks from both layers. The blue arrow indicates the interference between X-rays diffracted from both layers. The Bragg diffraction peak shift indicated by the red arrow implies the presence of tensile strain in the film layer. (**c**) Time-dependent angular shifts in Bragg peak position at early times following laser excitation. The initial rapid variations are due to the formation and propagation of an impulsive strain wave. (**d**) The same angular shifts but tracked over a longer, logarithmic timescale for both heterostructure layers. Both lattices relax towards their equilibrium positions after several microseconds.

To evaluate the thermal resistance within our multi-layered system, we measured the temporal progression of lattice strain across each layer [56]. Figure 1a depicts our experimental setup and the methodology for quantifying thermal resistance. The experiment was conducted at the 7ID-C insertion device beamline at the Advanced Photon Source (APS) [47,53]. We used 10 KeV X-rays monochromatized by a water-cooled double-crystal diamond (111) monochromator. The X-rays were focused horizontally using a KB mirror, producing a beam spot size of 50 μm^2^ at the sample’s location. The sample was placed at the center of a four-circle diffractometer, allowing us to resolve a narrow X-ray rocking curve with 0.5 millidegree angular resolution [47]. An 800-nm wavelength near-infrared pump beam with a 100-fs pulse duration was generated from an amplified Ti:sapphire laser system. This was synchronized with the APS storage ring’s revolution frequency. After frequency doubling to 400 nm, the laser beam was aligned with the X-ray beam on the sample’s surface. The relative time delay between the X-ray and laser pulses was controlled electronically, with a timing jitter of less than 2 ps. The TRXD measurement’s temporal resolution was approximately 100 ps full-width half maximum (FWHM) [47].

Our sample was a 500 nm-thick (0 0 1) Al${}_{0.3}$Ga${}_{0.7}$As film on a 500 μm (0 0 1) GaAs substrate, prepared using molecular-beam epitaxy [46]. The aluminum composition of 0.3 ensured a minimal lattice mismatch between the film and substrate. The optical pump’s absorption depth in the film was limited to 100 nm. We measured transient changes in the symmetric (0 0 4) Bragg diffraction peaks of the AlGaAs film and GaAs substrate as functions of the delay between the optical and X-ray pulses [56]. The diffracted X-rays were collected using a high-speed Avalanche Photodiode (APD) in proportional detection mode, paired with a digital oscilloscope. As the sample cooled due to heat transfer across the epitaxial interface, the time-dependent strain provided a direct measurement of temperature variations in both layers on a nanosecond timescale [57].

## 3. Results and Discussion

### 3.1. X-ray Diffraction Analysis

Figure 1b presents the X-ray diffraction curves from the symmetric (0 0 4) Bragg reflection of the laser-excited AlGaAs/GaAs heterostructure at specific time intervals post-optical excitation at an absorbed fluence of 3 mJ/cm${}^{2}$. The two distinct rocking curve features from the AlGaAs film (highlighted with a green background) and the GaAs substrate (gray background) are clearly discernible. The angular separation between these features confirms a lattice mismatch of less than 0.1% along the growth direction between the layers prior to laser excitation. The oscillatory intensity fringe pattern between these peaks arises due to interference between X-rays diffracted from the sidebands of the film and substrate peaks. The film thickness, estimated to be 500 nm based on the oscillation period, aligns with the sample design. The high fringe contrast $\beta =\frac{I_{\max}\;-\;I_{\min}}{I_{\max}\;+\;I_{\min}}$ before laser excitation suggests an atomically sharp interface between the two materials. Under the kinematical diffraction approximation, shifts in the Bragg peak correspond to changes in the lattice parameter, averaged across the X-ray extinction depth. The strain can be derived from the angular shift using the relation:

(1) $$\lambda =2\left(d\pm \Delta d\left(\Delta t\right)\right)\sin\left(\theta_{B}\pm \Delta \theta_{B}\left(\Delta t\right)\right)$$

where λ is the X-ray wavelength, and d(Δt) and $\theta_{B}\left(\Delta t\right)$ represent the temporally evolving crystal lattice spacing and Bragg diffraction angle, respectively [52]. This deviation provides insights into the averaged changes in lattice spacing for both the film and substrate layers. By tracking the Bragg peak centroid shifts over time, we can precisely quantify the strain migration in the depth direction.

### 3.2. Transient Structural Dynamics

Post-laser excitation, we observe immediate shifts of the Bragg diffraction peaks towards smaller angles for the film, as indicated by the red arrow in Figure 1b (bottom). This shift suggests the development of tensile strain near the AlGaAs film surface. Such rapid structural changes in early time scales (Δt < 30 ns) are attributed to the generation and propagation of longitudinal elastic waves or ballistic phonons [27,45,52]. The transient spatial distribution of these acoustic waves results in a significant strain gradient throughout the X-ray probe depth. This gradient is evident from the distorted intensity fringe pattern observed in the early time scales in Figure 1b (top). The reduced fringe contrast indicates a loss of structural ordering at the interface. At longer time delays (Δt ≥ 60 ns), the Bragg diffraction peak from the film layer begins to shift towards larger angles, indicating heat transfer from the film layer to the substrate.

### 3.3. Interface Distortion and Recovery

**Figure 2:**
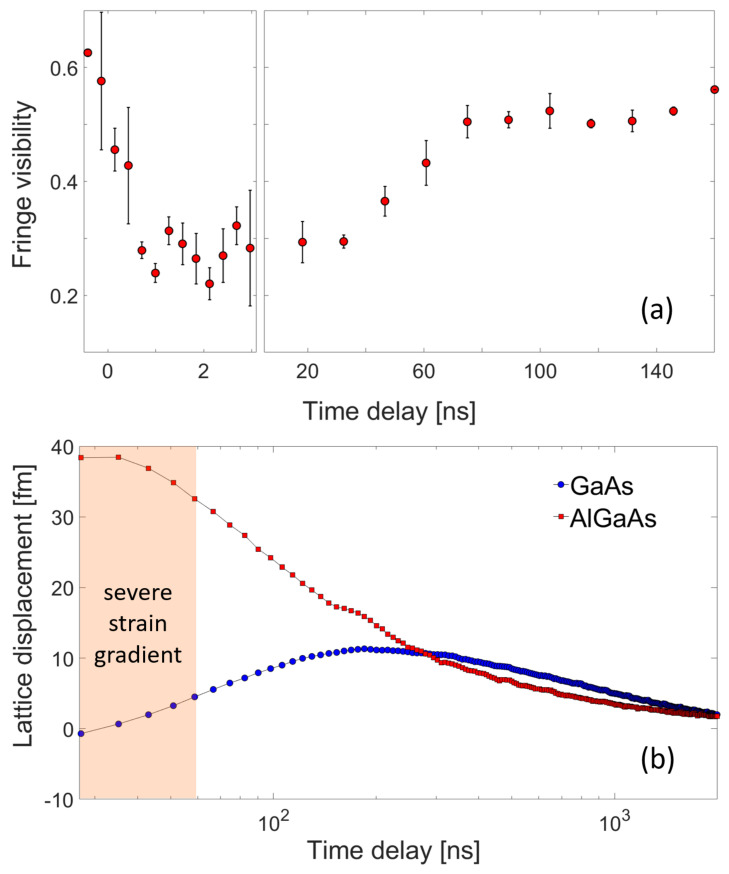
(**a**) The temporal evolution of interference fringe visibility within the initial 60 ns time delay suggests the existence of a significant strain gradient within the x-ray probe depth. (**b**) Shifts of the diffraction peak centroids obtained via Gaussian fitting are used to determine lattice displacements in both the film and substrate along the surface-normal direction as a function of time.

**Figure 3:**
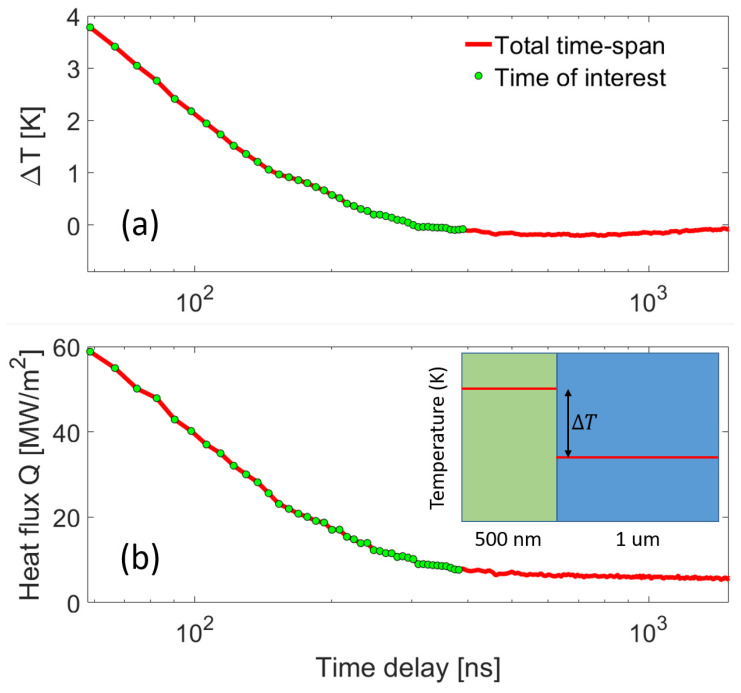
(**a**) The average layer strains are translated into temperatures by applying thermal expansion coefficients. This allows us to determine the time-dependent temperature difference, denoted as ΔT, between the film and substrate layers. (**b**) Calculated heat flux across the heterostructure interface as a function of time.

The epitaxial interface undergoes significant distortion, as indicated by the transient reduction in interference fringe visibility. In Figure 2a, the visibility contrast drops from 0.6 to 0.2 within the initial 3 ns and remains consistent until approximately 60 ns. This period, labeled as ‘severe strain gradient’ in Figure 2b, complicates the precise estimation of average lattice displacements without comprehensive strain modeling [53]. The recovery of fringe contrast at longer time delays suggests uniform heating of both the film and substrate. For thermal transport analysis, we focus on the TRXD data sets obtained after the 60 ns time delay. Assuming local thermal equilibrium for each layer, we converted the lattice strain into temperature using Bragg’s law and the bulk linear thermal expansion coefficients for the substrate and film. Figure 3a displays the temperature difference between the film and substrate layers over time. Notably, a measurable temperature difference is observed at the epitaxial interface, highlighting the presence of a non-negligible thermal boundary resistance, often overlooked in previous studies [46,56].

### 3.4. Heat Flux Evaluation

**Figure 4:**
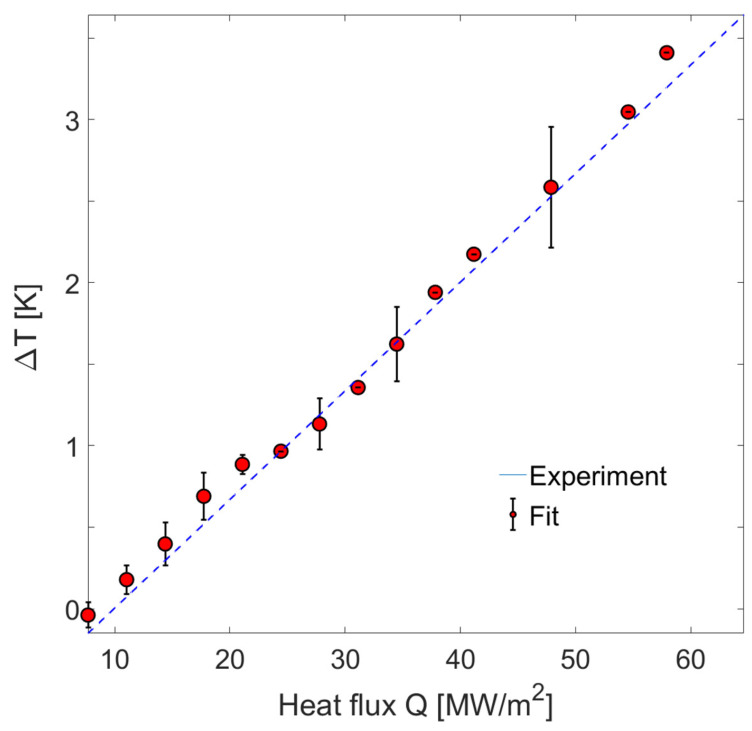
Measured temperature difference across the interface as a function of heat flux. The slope of the fitted line yields a measurement of the thermal resistance of the heterostructure sample.

From the transient temperature reduction in the film layer, we can determine the magnitude of heat flux per unit area moving toward the substrate layer. The heat flow across the interface is modeled as a 1-dimensional problem due to the large pump laser spot size compared to the X-ray probe area. The heat transfer rate is given by:

(2) $$Q_{\mathrm{AlGaAs}}\left(t\right)=\frac{C_{p}\times \rho \times dz\times dT\left(t\right)}{A\times dt}$$

where *A* is the heat transfer area, dt is the measurement time interval, and dT(t) is the time-dependent temperature difference between the layers. Figure 3b illustrates the calculated heat transfer rate per unit area from the film to the substrate over time. The bulk material properties used in the analysis are summarized in Table 1. In Figure 4, plotting the temperature difference against the heat flux reveals a linear relationship. The slope of this relationship represents the total thermal resistance of our heterostructure system. Our results indicate an effective thermal resistance of $\left(6.43\pm 0.14\right)\times 10^{-8}\;m^{2}K/W$.

### 3.5. Fourier’s Law and Thermal Boundary Resistance

The linear relationship between heat flux and temperature drop suggests effective phonon transport in our system adhering to Fourier’s law of diffusion. Assuming a simple three-layer system, the overall heat flow is subject to three thermal resistances: conduction resistance for each layer and thermal boundary resistance. Based on the typical bulk thermal conductivity values [59,60,61], we estimate that the thermal resistance for AlGaAs film and GaAs substrate layers probed by the X-rays is $3.57\times 10^{-8}\;m^{2}K/W$ and $2.73\times 10^{-8}\;m^{2}K/W$, respectively. The total expected thermal resistance of $6.30\times 10^{-8}\;m^{2}K/W$ is very close to our experimental value. Subtracting the expected thermal resistance from our experimental result yields a residual resistance of $1.31\times 10^{-9}\;m^{2}K/W$, which we attribute to the thermal boundary resistance (TBR) at the epitaxial interface. Table 1 shows our experimental results for the estimated thermal boundary resistance at the interface between the epitaxially grown film and the semiconductor substrate. Our findings align with previously reported TBR values for AlGaAs/GaAs heterostructures [54,55,58] and provide direct structural evidence for the TBR at a perfect atomic-level interface, where TBR exists due to mismatches in material properties on either side of the interface rather than due to the characteristics of the interface itself. Ultimately, the overall picture of nanoscale thermal transport is incomplete without proper partitioning of energy at the heterostructure interfaces.

## 4. Conclusions

In this study, transient lattice displacement measurements were employed to ascertain the temperature variations within each layer of the AlGaAs/GaAs heterostructure. Through detailed analysis of the temperature changes in the film layer, we quantified the heat flux emanating from this layer. By correlating these temperature changes with time, we were able to calculate the heat flux values, thereby enhancing our understanding of the thermal dynamics within the heterostructure. Our results indicate that the heat flux responds linearly to the temperature drop across the film–substrate interface, suggesting that thermal diffusion in our system is primarily a diffusive process. Additionally, we estimated the thermal boundary resistance at the epitaxial interface, which appears to constitute approximately 2% of the total thermal resistance in this heterostructure system. This finding highlights the interface’s role in overall thermal transport.

The substantial heat dissipation in newly integrated circuits of miniaturized electronic devices underscores the imperative for meticulous cooling design and precise thermal analysis. This is essential to prevent potential malfunctions caused by hot spots, which can be detrimental to the functionality of these devices. Our study contributes to this goal by providing a detailed understanding of thermal resistances, which are influenced by material thermal conductivity and the interactions between various film materials. Notably, the intricate nature of thermal contact resistance, as revealed in our findings, poses a significant challenge in thermal management [62].

Furthermore, the implications of our research extend beyond the realm of electronic devices. They are also pertinent to electronic packaging, aerospace technology, and optoelectronic devices, where managing thermal contact resistance is crucial. In critical environments such as nuclear power plants, where efficient heat transfer through thin-film materials is vital, our insights could be instrumental for identifying and mitigating thermal design flaws [63].

In summary, our study not only elucidates the thermal dynamics of AlGaAs/GaAs heterostructures but also provides a robust framework for future investigations into similar systems. The insights gained are particularly relevant for improving thermal management in various high-tech applications, highlighting the need for a comprehensive approach to understanding and managing thermal conductance at the microscopic level.

## Figures and Tables

**Table 1:** Bulk material parameters for the heat flux evaluation and comparison of thermal boundary resistance values with previous studies.

Materials	Al${}_{0.3}$Ga${}_{0.7}$As	GaAs
Thermal expansion coefficient ($\times 10^{6}$ )	5.57	5.75
ρ (g/cm${}^{3}$)	4852	5320
$C_{p}$ (J g${}^{-1}$ K${}^{-1}$)	356	320
$k_{s}$ (W m${}^{-1}$K${}^{-1}$)	55.0	13.2
Method	Current work	Previous studies
TBR ($\times 10^{9}$ Wm${}^{-2}$K${}^{-1}$)	1.30	1.3 [55], 1.4 [58], 2∼8 [54]

## Data Availability

The data presented in this study are available on request from the corresponding author.
